# Twelve quick steps for genome assembly and annotation in the classroom

**DOI:** 10.1371/journal.pcbi.1008325

**Published:** 2020-11-12

**Authors:** Hyungtaek Jung, Tomer Ventura, J. Sook Chung, Woo-Jin Kim, Bo-Hye Nam, Hee Jeong Kong, Young-Ok Kim, Min-Seung Jeon, Seong-il Eyun

**Affiliations:** 1 School of Biological Sciences, The University of Queensland, St Lucia, Queensland, Australia; 2 Centre for Agriculture and Bioeconomy, Queensland University of Technology, Brisbane, Queensland, Australia; 3 Genecology Research Centre, School of Science and Engineering, University of the Sunshine Coast, Sippy Downs, Queensland, Australia; 4 Institute of Marine and Environmental Technology, University of Maryland Center for Environmental Science, Baltimore, Maryland, United States of America; 5 Genetics and Breeding Research Center, National Institute of Fisheries Science, Geoje, Korea; 6 Biotechnology Research Division, National Institute of Fisheries Science, Busan, Korea; 7 Department of Life Science, Chung-Ang University, Seoul, Korea; University of Toronto, CANADA

## Abstract

Eukaryotic genome sequencing and de novo assembly, once the exclusive domain of well-funded international consortia, have become increasingly affordable, thus fitting the budgets of individual research groups. Third-generation long-read DNA sequencing technologies are increasingly used, providing extensive genomic toolkits that were once reserved for a few select model organisms. Generating high-quality genome assemblies and annotations for many aquatic species still presents significant challenges due to their large genome sizes, complexity, and high chromosome numbers. Indeed, selecting the most appropriate sequencing and software platforms and annotation pipelines for a new genome project can be daunting because tools often only work in limited contexts. In genomics, generating a high-quality genome assembly/annotation has become an indispensable tool for better understanding the biology of any species. Herein, we state 12 steps to help researchers get started in genome projects by presenting guidelines that are broadly applicable (to any species), sustainable over time, and cover all aspects of genome assembly and annotation projects from start to finish. We review some commonly used approaches, including practical methods to extract high-quality DNA and choices for the best sequencing platforms and library preparations. In addition, we discuss the range of potential bioinformatics pipelines, including structural and functional annotations (e.g., transposable elements and repetitive sequences). This paper also includes information on how to build a wide community for a genome project, the importance of data management, and how to make the data and results Findable, Accessible, Interoperable, and Reusable (FAIR) by submitting them to a public repository and sharing them with the research community.

## Introduction

Genome projects employ state-of-the-art DNA sequencing, mapping, and computational technologies (including cross-disciplinary experimental designs) to expand our knowledge and understanding of molecular/cellular mechanisms, gene repertoires, genome architecture, and evolution. The revolution in new sequencing technologies and computational developments has allowed researchers to drive advances in genome assembly and annotation to make the process better, faster, and cheaper with key model organisms [1,2].

Such technical advantages and established recommendations and strategies have been widely applied in humans [3–6], terrestrial animals [7–12], and plants and crops [13–18]. Genomic applications in aquatic species that could be potentially important for aquaculture are slower compared with human, livestock, and crops [19–21], compounded by larger diversity, lack of reference genomes, and more novice aquaculture industries. Given that aquaculture is the most rapidly expanding food sector, with the widest diversity of species cultured, it is poised for rapid adoption of genomics applications as these become more accessible. For any specific advice on application of genomics to aquaculture, please refer to previous works [19–25].

Before genome sequencing, a must-have step involves RNA sequencing (RNA-seq) that has provided significant insights into the biological functions [26–30]. RNA-seq plays a key role in genome annotation [31–36] through the identification of protein-coding genes based on transcriptome sequencing data and ab initio or homology-based prediction. However, the use of RNA-seq for genome assembly is limited to genome scaffolding [37]. While RNA-seq is a powerful technology that will likely remain a key asset in the biologist’s toolkit, recent single-molecule mRNA sequencing approaches (e.g., Pacific Bioscience [PacBio] and Oxford Nanopore Technology [ONT]) have provided significant improvements in gene and genome annotation, making them appealing alternatives or complementary techniques for genome annotation [38–40].

Restriction site–associated DNA sequencing and diversity array technology are cost-effective methods that mainly focus on the detection of loci and the segregation of variants or genome-wide single nucleotide polymorphisms. The generation of genetic linkage maps has been successfully applied to recognize key components in the sustainable production of aquaculture species [41,42]. These attempts have resulted in the emphasis of genomic evaluations/selections or advanced selective breeding programs for desirable traits, such as growth, sex determination, sex markers, and disease resistance [42]. While these inexpensive techniques have been powerful tools for understanding the genetics of adaptation, recent studies have indicated their limitations for genome scans because they will likely miss many loci under selection, particularly for species with short linkage disequilibrium [43]. However, the widespread use of whole-genome sequencing (WGS) allows the detection of a full range of common and rare/hidden genetic variants of different types across almost entire genomes.

Many seminal biological discoveries in the 20th century were made using only a genetic analysis of a few selected model organisms because they were readily available for genetic analysis [44]. However, a high-quality and well-annotated genome assembly is increasingly becoming an essential tool for applied and basic research across many biological disciplines in the 21st century that can turn any organism into a model organism. Thus, securing more complete and accurate reference genomes and annotations before analyzing post-genome studies such as genome-wide association studies, structural variations, and posttranslational studies (methylation or histone modification) has become a cornerstone of modern genomics. Chromosome-level high-quality genomes (including structural and functional annotations) are differentiated from draft genomes by their completeness (low number of gaps and ambiguous *N*s), low number of assembly errors, and a high percentage of sequences assembled into chromosomes. Advances in next-generation sequencing (NGS) technologies and their analytical tools have made assembling and annotating the genomic sequence of most organisms both more feasible and affordable [33,45,46]. Table 1 shows recent chromosome-level genome assemblies and provides a rough estimate of the sequencing depth and costs for beginners to achieve a chromosome-level genome assembly. For diploids, using a minimum 60× depth for PacBio, ONT, 60× for Illumina (San Diego, California, United States of America), and 100× for Hi-C data (Phase Genomics, Seattle, Washington, USA) (an extension of chromosome conformation capture, 3C) is recommended. High-quality end-to-end genome assembly and annotation of small eukaryotic (approximately 1 Gb diploid) and prokaryotic organisms have been achievable with small-to-medium financial resources and limited time, labor, and skill commitments. Nearly all eukaryotic genomes still represent a significant challenge for most aquatic species that have large and complex genomes and no reference genomes.

**Table 1:** Summary of recently published chromosome-level genome assemblies in aquaculture species using long-read sequences^a^^,^^b^.

Scientific name	GS (Gb)	Final output			Input detail and depth (×)					BAs	Reference
		AGS (Gb)	FSN	N50 (Mb)	IM	PacBio	ONT	10xGC	Hi-C (×)		
Fish											
*Collichthys lucidus*	0.83/DP	0.88	24	1.1	63	109			233	Sex determination genes and chromosomes	[72]
*Clupea harengus*	0.81/DP	0.79	26	29.85	50	76			20	Chromosome rearrangement and spawning time	[10]
*Epinephelus akaara*	1.11/DP	1.14	24	46.03	49		96		100	Chromosome-level reference genome	[73]
*Epinephelus lanceolatus*	1.07/DP	1.09	24	46.2	134				0.2	Innate immunity and growth	[74]
*Sebastes schlegelii*	0.87/DP	0.81	24	3.85	132	66			189	Maternal reproductive system	[75]
*Lateolabrax maculatus*	0.65/DP	0.67	24	22.34	321				109	Chromosome-level reference genome	[41]
*Oplegnathus fasciatus*	0.78/DP	0.77	24	33.5	116	80			118	Chromosome-level reference genome	[43]
*Pelteobagrus fulvidraco*	0.72/DP	0.73	26	25.8	70	53			200	Chromosome-level reference genome	[40]
Shellfish											
*Sinonovacula constricta*	1.33/DP	1.22	19	65.93	148	148	136	123	154	Chromosome-level reference genome	[76]

^a^This table represents a selection of recent aquaculture genome works focusing on whole-genome assemblies using BioNano and/or Hi-C data (at least 1 technology used) since 2018. In addition, the table does not include any pure TGS/SGS/hybrid genome assemblies without BioNano/Hi-C data, single-cell sequencing, or transcriptomes. If the original report had no estimated input depth, this was calculated from the raw data. For the most recent global statistics, we highly recommend visiting the associated GenBank BioProject.

^b^AGS, assembled genome size; BAs, biological applications; DP, diploid; FSN, final scaffold number (pseudochromosome number); GS, genome size; IM, Illumina (combined paired-end [PE] and mate-pair [MP] reads); ONT, Oxford Nanopore Technology; PacBio, Pacific Bioscience; SGS, second-generation sequencing; TGS, third-generation sequencing; 10xGC, 10x Genomics Chromium.

Furthermore, the following fundamental questions should be addressed: Why are genome projects or WGS necessary? What is the aim of a genome project? What kind of information is the research community expected to gather? Even from the beginning of a genome project, describing the expected end product, including project duration/budget, chromosome end-to-end completion, genome browser, and research paper, is required. In particular, if budget is a major obstacle, the best option to raise funds to support the genome project (e.g., industry or government support) must be determined. In addition to the abovementioned limitations, another essential element is bioinformatics, which has become a common denominator to produce and use software that can be applied to biological data in different contexts. As big data and multi-omics analyses are becoming mainstream, computational proficiency and literacy are indispensable skills in a biologist’s toolkit in modern scientific society. All “omics” studies require a certain degree of computational biology: The implementation of analyses requires programming skills and knowledge of computer languages, while experimental design and interpretation require a solid understanding of analytical approaches [47,48]. These could be daunting tasks for biologists who are unfamiliar with computational standards (e.g., codes, pipelines, and system environments) and resources (e.g., SourceForge, Bitbucket, GitLab, and GitHub). While academic cores, commercial services, and collaborations can aid in the implementation of analyses, the computational literacy required to design and interpret omics studies cannot simply be replaced or supplemented [47,48].

In the absence of a standard approach for genome projects, this paper aims to provide practical steps to facilitate project completion before embarking upon a genome assembly and annotation project (mainly for eukaryotic genomes). The target audience is anyone entering this field for the first time, particularly those who do not specialize in genomics research. While we can strive to answer questions in a manner that considers the beginner’s perspective, certain aspects (e.g., assembly algorithms and computer environments) might require further reading for an in-depth understanding.

### Step 1: Build a wide community for the project if possible

All genome projects have a common but monumental goal: sequencing the entire target genome for a wide range of genomics applications. While genomics is a rich field, one of the most prominent scientific objectives is probably securing the future of sustainable food sources by harnessing the power of genomics (i.e., desirable traits) [19–21,23–25], particularly for agriculture. If the species of interest is distinct from the wild, cultured, or harvested, it necessitates networking and building a scientific or stakeholder community to support the project. This usually requires a multi-institutional effort to both initiate and—more importantly—complete the genome project and then interpret the vast quantities of sequencing information produced for any given organism. As expected, WGS/genome projects’ infrastructure demands are particularly high as varying interpretations may require facilities, personnel (skill intensive), and software (knowledge intensive) that suit the needs of immediate analyses, ongoing reanalyses, and the integration of genomic and other phenotype information (or desirable traits). Data storage, maintenance, transfer, and analysis costs will also likely remain substantial and represent an increasing proportion of overall sequencing costs in the future. Moreover, professional groups (including students), expert panels, and field farmers acknowledge that there is a need for educational programs specific to WGS demands. Addressing these needs will likely require substantial investment by agriculture production care systems. Thus, the real cost of WGS—including ongoing maintenance—could be even higher. Despite these burdens, most genome projects bring together leading researchers to work together and build large datasets of DNA from target genomes, which has significantly benefited the research community. These efforts facilitate the sharing of sequence data and help research advance. In particular, smaller research groups that have less experience and are poorly equipped in areas including raw read sequencing and assembly and annotation should consider the main features and steps outlined here via community collaboration. In the case of funding for genome projects, applying for government grants and receiving corporate sponsorships as a consortium could be considered potential solutions as these avenues have been successful for humans, livestock (cow, pig, and sheep), crops (Arabidopsis, rice, and tomato), and aquaculture (salmon, oyster tilapia, and prawn).

### Step 2: Gather information about the target genome

Every genome sequencing, assembly, and annotation project is different due to each subject genome’s distinctive properties. There are four fundamental aspects that must be considered when embarking on a new genome project: the genome size, levels of ploidy and heterozygosity, GC content, and complexity. These will directly affect the overall quality and cost of genome sequencing, assembly, and annotation [14,49].

How big is the genome? The genome size will greatly influence the amount of data that must be ordered and analyzed. To assemble a genome, securing a certain number/amount of sequences/depth/coverage (called reads) is the first step before proceeding with ordering sequence data. To get an idea of the size and complexity of a genome, publicly available databases for approximate genome sizes are accessible for fungi (http://www.zbi.ee/fungal-genomesize), animals (http://www.genomesize.com), and plants (http://data.kew.org/cvalues). Selecting a closely related species is a practical option if the information on a target species is unavailable from a public database. Alternatively, the two widely used flow cytometry and *k*-mer frequency distribution methods could provide reliable genome size estimates to predict repeat content and heterozygosity rates. Flow cytometry is a fast, easy, and accurate system of simultaneous multiparametric analysis for nuclear DNA content including a ploidy level that isolates nuclei stained with a fluorescent dye [50,51]. *K*-mer frequency distribution, a pseudo-normal/Poisson distribution around the mean coverage in the histogram of *k*-mer counts, is a powerful and straightforward approach to use raw Illumina DNA shotgun reads to infer genome size, data preprocessing for de Bruijn graph assembly methods (tune runtime parameters for analysis tools), repeat detection, sequencing coverage estimation, measuring sequencing error rates, and heterozygosity [52,53]. It is highly recommended to use both flow cytometry and *k*-mer methods—the gold standard for genome size measures when designing genomic sequencing projects—because no single sequence-based method performs well for all species, and they all tend to underestimate genome sizes [54]. Is it a diploid, polyploid, or highly heterozygous hybrid species? If possible, it is better to use a single individual and sequence a haploid, highly inbred diploid organism [20,23,55], or isogenic line [56] because this will essentially minimize potential heterozygosity problems for genome assembly. While most genome assemblers are haploid mode (some diploid-aware mode) to collapse allelic differences into one consensus sequence, using complex polyploid or less inbred diploid genomes can greatly increase the number of present alleles, which will likely result in a more fragmented assembly or create uncertainties about the contigs’ homology [14,49]. If so, polyploid and highly repetitive genomes may require 50% to 100% more sequence data than their diploid counterparts [14].

Is there high/low GC content in a genomic region? Extremely low or high GC content in a genomic region is particularly known to cause problems for second-generation sequencing (SGS) technologies (also called short-read sequencing: mainly refer to Illumina sequencing), resulting in low or no coverage in those regions [57]. While this can be compensated for by increasing the coverage, we would recommend using third-generation sequencing (TGS) technologies (PacBio and ONT) that do not exhibit this bias [14,49].

How many repetitive sequences (or transposable elements) will likely be present in the genome? The amount and distribution of repetitive sequences, potentially occurring at different locations in the genome, can hugely influence genome assembly results, simply because reads from these different repeats are very similar and the assemblers’ algorithms cannot distinguish them effectively. This may eventually lead to misassembly and misannotation. This is particularly true for SGS reads and assemblies, and a high repeat content will often lead to a fragmented assembly because the assemblers cannot effectively determine the correct assembly of these regions and simply stop extending the contigs at the border of the repeats [58]. To resolve the assembly of repeats (or if the subject genome has a high repeat content), using TGS reads that are sufficiently long to include the unique sequences flanking the repeats is an effective strategy [14,49]. Thus, understanding the target genome and generating sufficient sequence data/read coverage is a crucial starting point in a genome assembly and annotation project.

### Step 3: Design the best experimental workflow

To meet the experimental goals and answer various biological questions, each application must come with different experimental designs. Above all, the development of high-quality chromosomally assigned reference genomes constitutes a key feature for understanding a species’ genome architecture and is critical for the discovery of the genetic blueprints for biologically significant traits. Once the reference genome has been completed, follow-up post-genome studies can be substantially completed with high accuracy.

While NGS is a useful tool for determining DNA sequences, certain parameters need to be considered prior to running an NGS experiment, such as quality control, SGS versus TGS, read length, read quality/error rate, number of reads, genome read coverage/depth, library preparation, and downstream applications. Recent papers have provided useful recommendations and strategies to ensure the success of NGS experiments by selecting the correct products/technologies and methods for the project [14,59–61]. If money is no obstacle, using TGS data (PacBio and ONT) and Hi-C data is recommended [14], which are also widely accepted approaches for reaching a chromosome-level genome assembly (Table 1) for aquaculture or any other species. While a hybrid approach using Illumina/10x Genomics Chromium (10xGC) and Hi-C data has been proposed as a cost-effective method, this approach’s contiguity could be lower than that of the combination of TGS data and Hi-C data [14].

Another important point to consider is whether genome assembly should be de novo or reference guided/assisted (Table 2). De novo assembly is the most widely adopted, but when complete genomes of closely related species are available, reference-guided/assisted genome assembly could be an attractive option because of its lower requirements for coverage data and computational memory [14]. However, early works have warned against its applications in genome assembly because the resultant assemblies may contain biases toward errors and chromosomal rearrangements in the existing reference genome [62–64]. No matter which assembly approaches and technologies are taken, genome assembly’s purpose is to construct a consensus haploid or haploid-phased chromosome-level assembly. Most extensively used genome assemblers typically collapse the 2 sequences into 1 haploid consensus sequence and thus fail to capture the diploid nature of target organisms. While this has been a key challenge in the bioinformatics and biology community, recent works have demonstrated the effectiveness of generating accurate and complete haplotype-resolved assemblies for diploid and polyploid species (Table 2). While we have provided a brief summary of commonly used tools (Table 2), the comprehensive program list focused on TGS reads can be accessed at LRS-DB (https://long-read-tools.org). Thus, selecting the appropriate tools and pipelines is important to achieve accurate chromosome-scale assemblies in a timely manner by leveraging speed and sensitivity in the contiguity and quality of genome assemblies.

**Table 2:** Commonly used tools and programs for genome assembly.

Name	Official link	Main feature
De novo genome assemblers for TGS reads		
Falcon/HGAP	https://pb-falcon.readthedocs.io/en/latest/#	Diploid-aware mode including trim, correction, and consensus for PacBio reads
CANU	https://github.com/marbl/canu	A fork of the Celera Assembler including trim, correction, and consensus for TGS reads
SMARTdenovo	https://github.com/ruanjue/smartdenovo	De novo assembler including all-vs.-all raw read alignments without an error correction stage for TGS reads
MECAT	https://github.com/xiaochuanle/MECAT	Ultrafast mapping, error correction, and de novo assembly tools for single-molecule sequencing reads
Flye	https://github.com/fenderglass/Flye	A repeat graph mode including trim, correction, and consensus with polishing for TGS reads
Shasta	https://github.com/chanzuckerberg/shasta	A run-length representation of ONT reads
De novo genome assemblers for SGS reads		
ABySS2	https://github.com/bcgsc/abyss	An assembler intended for SGS PE and linked-reads
AllPath-LG	http://software.broadinstitute.org/allpaths-lg/blog/	Uses a unipath graph from the *k*-mer paths to collapse repeats
MEGAHIT	https://github.com/voutcn/megahit	An ultrafast and memory-efficient assembler for SGS reads
SOAPdenovo	http://soap.genomics.org.cn	De Bruijn graph assembler with an error correction stage
De novo genome assemblers for hybrid reads		
MaSuRCA	https://github.com/alekseyzimin/masurca	An assembler combining the benefits of the de Bruijn and Overlap-Layout-Consensus assembly approaches for SGS and TGS reads
Reference-guided/assistance assemblers		
Ragout	https://github.com/alekseyzimin/masurca	Chromosome-level scaffolding
RaGOO	https://github.com/malonge/RaGOO	Pseudochromosome construction
RGAAT	https://github.com/wushyer/RGAAT_v2	Genome assembly and annotation
Haplotype/phase assemblers		
Falcon-Unzip	https://pb-falcon.readthedocs.io/en/latest/index.html	PacBio reads
Falcon-Phase	https://github.com/phasegenomics/FALCON-Phase	PacBio reads
Triobinning	https://github.com/skoren/triobinningScripts	ONT reads
Platanus-allee	http://platanus.bio.titech.ac.jp/platanus2	SGS and TGS reads
WhatsHap	https://bitbucket.org/whatshap/whatshap/src/master/	SGS and TGS reads
IntegratedPhasing	https://github.com/vibansal/IntegratedPhasing	SGS and TGS reads
HaploConduct	https://github.com/HaploConduct/HaploConduct	SGS and TGS reads
HaplotypeAssembler	https://github.com/ComputationalGenomics/HaplotypeAssembler	SGS and TGS reads

ONT, Oxford Nanopore Technology; PacBio, Pacific Bioscience; PE, paired-end; SGS, second-generation sequencing; TGS, third-generation sequencing.

### Step 4: Choose the best sequencing platforms and library preparations

To sequence an organism’s entire genome (WGS), it must be prepared into a sample library from high-quality genomic DNA. A library is a collection of randomly sized DNA fragments that represent the sample input; its size can vary depending on the choice of sequencing technology. Sample library preparation for WGS is dependent on two considerations: (1) the genome size of the target sample organism; and (2) the amount of sample available to be sequenced. Given the vast range of library preparation products, we can only provide general suggestions for library preparations. For more platform-specific library preparation and sequencing guides, refer to the vendor’s products and/or services page. The recommended procedure is to select the best and most cost-effective library preparation and sequencing technology after considering the given research goal and budget.

The rapid adoption of WGS has been facilitated by the development of SGS and TGS technologies, which have dramatically reduced sequencing costs and simplified genome assembly. It is possible to select short (Illumina, 454, SOLiD, and Ion Torrent), long (ONT and PacBio), or a combination (hybrid) read. Comprehensive guidelines (including pros and cons) for selecting the correct sequencing technology have been extensively described in previous works [14,59,61,65]. Briefly, while SGS technologies can produce high-throughput, fast, cheap, and highly accurate reads of lengths in the range 75 to 700 bp, they show limited ability to resolve complex regions with repetitive or heterozygous sequences, which results in incomplete or heavily fragmented genome assemblies. According to Illumina, widely used SGS technology—the TruSeq PCR-free Library Preparation Kit—is ideal for any size of genome with a large sample input if there is 2 μg of genomic DNA available. However, the Nextera DNA Library Prep Kit (Illumina) is perfect for large and complex genomes with a small sample input. Meanwhile, the TruSeq Nano DNA Library Prep Kit (Illumina) is ideal for any size genome with a small sample input if there is only 200 ng of genomic DNA available. However, the Nextera DNA XT DNA Library Preparation Kit (Illumina) is perfect for small genomes, plasmids, and amplicons. Additional Illumina library preparation methods and sequencing platforms for high throughput have been extensively reviewed [66,67].

Meanwhile, TGS technologies can produce long single-molecule reads (averaging >30 kb) with complete contiguity, facilitating assembly. However, long-read technologies suffer from both high costs per base and high error rates. To overcome this disadvantage, the PacBio RS II or SEQUEL system (Pacific Biosciences, Menlo Park, California, USA) has been released that could generate 10 to 15 times more data than the original SEQUEL system with even more accurate long reads (HiFi reads could be ABI Sanger quality up to 40 kb). According to PacBio, the SMRTbell Template Prep Kit (Pacific Biosciences) with 20 to 40 kb template preparation using BluePippin Size Selection is recommended for WGS [14,68]. For ONT, a combination of ligation sequencing, PCR sequencing, and rapid sequencing has been optimized for WGS [60,69]. In particular, the Rapid Sequencing Kit (SQK-RAD004) could produce even higher read lengths and some reads could be >2 Mb [70].

Combining data from both SGS and TGS in a “hybrid approach/assembly” can compensate for the downsides of both approaches and is a cost-effective method because SGS data can correct errors in TGS reads [33,71–75]. Alternatively, the development of an advanced “hybrid” approach, such as incorporating 10xGC data or medium-size single-molecule DNA fragment selection and tagging before short-read sequencing, could be a practical strategy to increase the continuity and accuracy of long reads [14]. While recent studies have highlighted the efficacy and cost-effectiveness of 10xGC linked-reads in diploid aquatic species’ genomes [76–79], the utility of this technology for complex and/or polyploid aquatic species is still being investigated. According to 10xGC, the Chromium Genome Reagent Kit is ideal.

Regardless of the sequencing technology and approach (SGS, TGS, or hybrid), incomplete and/or unfinished assemblies can still occur (e.g., those with gaps and fragments). Thus, additional techniques such as optical mapping (BioNano, San Diego, California, USA) and chromatin association (Hi-C) are highly recommended to facilitate contig joining and genome assembly completion [46,80–83]. Use of the Hi-C method over BioNano has been observed in aquaculture species (Table 1). The most widely used kit is the Proximo Hi-C Kit provided by Phase Genomics (https://www.phasegenomics.com/hi-c-kits).

### Step 5: Select the best possible DNA source and DNA extraction method

The extraction of high-quality DNA is the most important aspect of a successful genome project. Given the potential breadth of aquaculture species, each with their own peculiarities, extracted high-molecular-weight DNA should be free of contaminants either from the subjected material itself or from the DNA extraction procedure (e.g., polysaccharides, proteoglycans, proteins, secondary metabolites, polyphenols/polyphenolics, humic acids, carbohydrates, and pigments). While recent publications and commercial kits have provided valuable guidance [84–86], DNA extraction methodologies can be explored and adapted along the lines provided by the literature. In general, the minimum DNA input is required for Illumina and 10xGC > 3 ng, PacBio > 20 μg, ONT > 1 μg, BioNano > 200 ng, and Dovetail > 5 μg [14]. Depending on the project budget and sequencing platform accessibility, SGS and/or TGS technologies can be considered; we recommend using TGS that can deliver DNA of average size >25 kb. Certain species (e.g., mollusks containing high levels of polysaccharide) warrant more careful planning than others. A modified low-salt cetyltrimethylammonium bromide extraction protocol has produced excellent quality DNA of high molecular weight that is free from contaminants and shearing [87]. Other important considerations are the heterozygosity rate, amplification, and presence of other tissues/organisms [14,49]. The heterozygosity rate can be reduced using a single individual for extraction. However, certain organisms require a pool of individuals to retrieve a sufficient amount of DNA, which will increase the genetic variability and lead to a more fragmented assembly. Attractive strategies include generating an inbred line of individuals for low-heterozygosity pooled sequencing and/or sequencing of haploid tissues as the foundation for filtering out paralogous sequence variants. These have been successful for cost-effective WGS and for optimizing the precision of allele and haplotype frequency estimates in aquaculture breeding [19,20,24,42,55]. When few cells are available, the genomic DNA must be amplified before sequencing, but this can often result in uneven coverage due to artificial effects (chimeric and/or fused unrelated sequences). The introduction of unwanted/unrelated organisms (e.g., contaminants and/or symbionts) and/or tissues (e.g., mitochondria and/or chloroplasts) should be minimized at the extraction and library preparation stages. This requires using tissue with a higher ratio of nuclear over organelle DNA because this can lead to higher coverage of the nuclear genome in the sequences. Whichever approach is adopted, there will be a need to refine the method to achieve several important quality metrics for genome sequencing.

Care should be taken for quality parameters (e.g., the chemical purity and structural integrity of DNA) and two recent works have made the recommendations outlined below for long-read technologies [14,49]. Generally, the measurement/quantification of purified DNA should be performed using both spectrophotometric and fluorescence-based methods (e.g., qubit). Samples with optical density (OD_260_:OD_280_) ratios of 1.8 to 2.0 are usually free of protein contamination. DNA concentrations at a 1:1 ratio (determined by spectrophotometry and fluorimetry, respectively) are very good indicators of whether they will be sequenced efficiently. To determine the integrity of DNA samples, contour-clamped homogeneous electric field or pulsed-field gel electrophoresis is appropriate when used with TapeStation or Fragment Analyzer (Agilent Technologies, Santa Clara, California, USA). Analyzing isolated DNA in this manner also facilitates decisions regarding shearing DNA to attain an optimal size range for sequencing. Thus, it is always worth investing time in getting high-quality DNA that will result in high-quality data and assembly to save time and money.

### Step 6: Check the computational resources and requirements

Installing open-source tools in one’s computational environment is not always either straightforward or trivial. It generally poses three potential problems: (1) the prerequisites of the tools created by diverse developers employing diverse programming frameworks differ; (2) the installation of various software items in one environment can lead to hard-to-resolve software dependency conflicts; and (3) upon successful installation, maintaining the environment and ensuring that all tools (including changes and updates) are working as expected remain difficult. Therefore, managing the data analysis environment becomes increasingly complex when a project requires many tools for genomic data analysis. While addressing the importance of the appropriate data and computing infrastructure to genome projects is difficult, the two following options (see Step 7: maximizing in-house workers or collaboration and outsourcing from the service provider) can be considered.

Access to high-performance computing or cloud-based computing systems is crucial for genome projects that require a large number of computing resources. As a general guide, the successful assembly of a moderately sized diploid genome (approximately 1 Gb) using software pipelines (Tables 1 and 2) requires a minimum computing resource of 96 physical central processing unit (CPU) cores, 1 TB of high-performance random-access memory (RAM), 3 TB of local storage, and 10 TB of shared storage [14]. However, the guide is scalable based on the amount of data, genome size, heterozygosity rate, and ploidy. Please note that runtimes, memory requirements, number of CPUs, and computational costs will increase geometrically because genome assembly is an all-by-all comparison. However, hard drive space to store raw and/or intermediate data (e.g., storage space) will increase linearly as the total amount/depth of coverage required does not dramatically change as genomes increase in size. In addition, the recommendations stated here will likely apply to larger and more complex genomes (e.g., crustaceans with numerous chromosomes) but at a slower rate and with higher computing resources and costs (obtaining more computing resources will increase costs). If participants’ or collaborators’ institutions are equipped with large in-house high-performance computing resources, they will likely have more direct access and practical assistance in their genome project. Otherwise, cloud-based computing is a potential solution that has been widely emphasized in previous works including easy-to-follow steps [88–90]. While cloud computing provides flexibility, competitive pricing, and continually updated hardware and software, it still requires assistance from information technology (IT) specialists to set up suitable cloud-based software. Thus, users should consider all possible options (including their research budget) to achieve the best outcome.

### Step 7: Choose the best computational design and pipeline

Optimizing a computational design and securing sufficient computer resources are essential steps to succeed in a genome assembly and annotation project. In addition, computational proficiency and literacy have become vital skills for biologists to design and interpret big data analyses and multi-omics studies [48]. Given the vast range of computational tools and requirements (different resource demands between assembly and annotation for each species), general suggestions are provided on the computational aspect. However, when establishing the best and most cost-effective computational design and requirement, it is important to consider three options: (1) maximizing in-house workers or collaboration; (2) outsourcing from a service provider; and (3) simulating data with different settings. Ultimately, the most suitable and practical approach in methodological computational biology research is recommended because there is no perfect computational design for genome assembly and annotation.

Before embarking on any actual data analyses, the overall goals should first be defined by understanding in-house workers and facilities because computational design requires extensive learning of computer and biology knowledge, which is a great challenge for most wet lab researchers/groups. If in-house workers and computer facilities are not ready to deliver successful outcomes, cross-disciplinary collaborations (computer science, data science, bioinformatics, and biology) could present great solutions. Initiating and successfully maintaining cross-disciplinary collaborations can be challenging but are highly rewarding because the combination of methods, data, and interdisciplinary expertise can achieve more than the sum of the individual parts alone [91].

Alternatively, work can be outsourced to a service provider. Outsourcing has the following benefits: (1) no need to hire more employees for computational design and analysis, which will reduce labor costs; and (2) there are more talents available at well-equipped companies that are very specialized in specific research fields. However, outsourcing also has the following disadvantages: (1) a lack of control as a contractor; (2) limited methods of communication (e.g., phone, e-mail, or online chat); and (3) the potential danger of poor quality work due to the inability to optimize pipelines (e.g., parameters) and outcomes.

No matter which approach is taken, the essential part is to have firsthand experience to select proper computational design and pipeline and to accurately interpret analyzed genome data. Due to its extensive range of analytical tools and application areas, employing an effective simulator (from the quality of raw reads to assembly evaluation) has become an essential step for benchmarking genomic and bioinformatics analyses [92–94]. In simulations, considering a (very) large number of datasets is generally not a problem, except when the analysis of each dataset is hugely computationally expensive (e.g., in the genome assembly stage). In practice, one should generate and analyze as many datasets as computationally feasible before embracing real empirical studies, particularly before undertaking real assemblies. In large genome assembly, simulating assemblies of down-sampled real data (e.g., 30× coverage/depth of genome) would be very useful for selecting the best pipeline and parameters without requiring too much computational time or cost. Ultimately, a simulation’s practical relevance depends on the similarity between the considered simulation settings and the real datasets in the area of application. The new method may be assessed in different ways depending on the context (e.g., by conducting simulations, applying the method to several real datasets, applying flexible parameter settings, and checking the underlying assumptions in practical examples). Therefore, simulations should not be limited to artificial datasets that correspond exactly to the assumptions underlying the new method as this would favor the new method [61,95–98].

### Step 8: Assemble the genome

**Fig 1:**
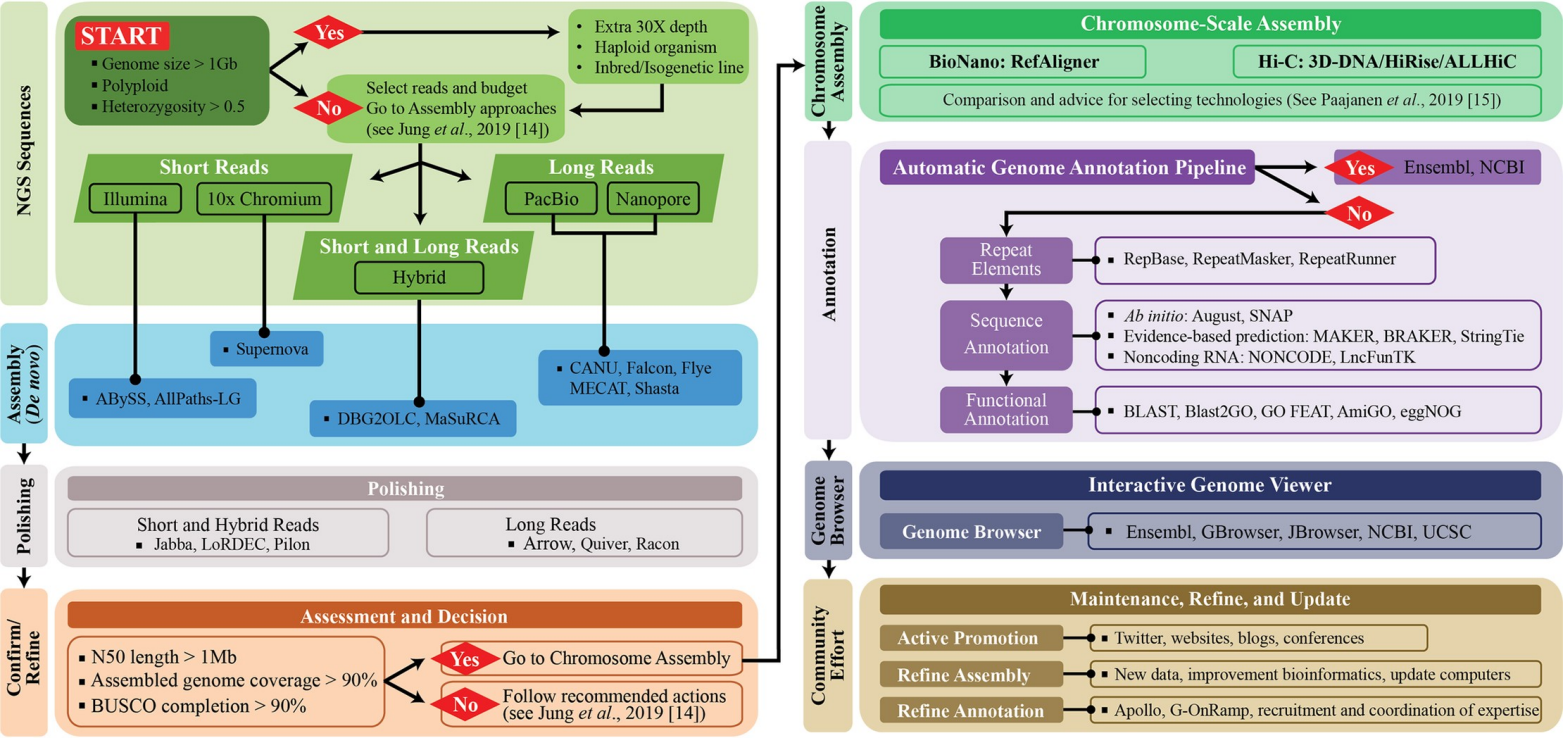
Recommended flowchart for genome assembly and annotation. NGS, next-generation sequencing.

Regardless of which pathway/strategy is chosen, the TGS approach is recommended over the SGS or hybrid approaches. In general, using multiple programs at each stage to predict the best assembly and annotation (Table 2) is also recommended because each approach and tool has limitations based on the problems inherent in the different algorithms and assumptions used. If the abovementioned steps (Steps 1–7) are met, the recommended flowchart and/or guideline for genome assembly, annotation, maintenance, and community effort would be as shown in Fig 1, which could be broadly applicable to any species. The rationale of each computational design, workflow, and decision tree is well described in Jung and colleagues [14], including the background information for each of their steps and the spectrum of available analytical options. Following the workflow and decision tree described by Jung and colleagues, the recommended tools herein are the TGS pipeline: PacBio/ONT read sequencing (remove all contaminated DNA; plastids/bacterial contamination) → read quality assessment, evaluation, and filtering → assembly → error correction and polishing using SGS reads → assessment → chromosome-level assembly using BioNano and Hi-C data. Several recent assemblies adopted from this pipeline (or similar) have shown notable improvements in the assembly of intergenic spaces and centromeres [33,72]. A potential assembly outcome from the new SEQUEL II (HiFi) reads would be even more promising (see Step 4) compared to its early version SEQUEL. In the SGS pipeline, if the target is a diploid organism, starting from 10xGC read sequencing over Illumina reads is ideal. Based on the results of the hybrid-based assemblies, the recommended pipeline starts from PacBio/ONT, and 10xGC read sequencing greatly helps build a highly accurate contiguous genome [78]. However, all assembly approaches/designs derived only from sequence reads will still contain misassemblies (inversions and translocations), these are mainly caused by the inability of both sequencing and assembly pipelines to cope with long tracts of repeat sequences or high levels of heterozygosity and polyploidization. Thus, using BioNano and Hi-C data is highly recommended for reaching chromosome-level assembly because these two methodologies/technologies can improve the assembly quality by validating the integrity of the initial assembly, correcting misorientations, and ordering the scaffolds.

### Step 9: Check the assembly quality before annotation

In the shotgun sequencing era, assembling a new genome mostly relies on computational algorithms and experimental designs (see Steps 6 and 7). The performance of such algorithms and designs, read lengths, insertion size of sequencing libraries, read accuracy, and genome complexity determines the accuracy and continuity of the genome assembly. Therefore, while estimating assembly quality is an unpredictable and challenging task that requires several statistical and biological validations, it remains an important step for a high-quality genome. Typically, the quality assessment for draft assemblies is carried out via statistical measurements and alignment to a reference genome (if available) [99]. These include overall assembly size (determining the match to the estimated genome size), measures of assembly contiguity (N50, NG50, NA50, or NGA50; the number of contigs; contig length; and contig mean length), assembly likelihood scores (calculated by aligning reads against each candidate assembly), and the completeness of the genome assembly (Benchmarking Universal Single-Copy Orthologs [BUSCO] scores and/or RNA-seq mapping) [100,101]. In computational biology, N50 is a widely used metric for assessing an assembly’s contiguity, which is defined by the length of the shortest contig for which longer and equal-length contigs cover at least 50% of the assembly. NG50 resembles N50 except for the metric, which relates to the genome size rather than the assembly size. NA50 and NGA50 are analogous to N50 and NG50 where the contigs are replaced by blocks aligned to the reference [99]. Thankfully, recent bioinformatics tools offer an automated pipeline to compute and evaluate the new genome quickly and accurately in a practical setting [44,102,103].

Additional strong indicators of quality include agreement with data on quantitative trait loci, expressed sequence tags (ESTs), fluorescent in situ hybridization experiments employing bacterial artificial chromosome clones, and the genome assembly’s contiguity with a chromosome-level genetic map. If the initial assembly attempt is unsatisfactory, three specific areas (contiguity, accuracy, and completeness) should be considered to determine the best path forward to improve the new assembly’s quality [14]. Generally, the best way to address high contig numbers with low average size is to acquire and incorporate more TGS or 10xGC (see Steps 3 and 4: hybrid assembly approaches) reads. When attempting to increase assembly quality, adding more and longer TGS reads tends to be more helpful for bridging existing contigs by increasing the size of the average contig; then, subsequently adding further BioNano and Hi-C data improves read accuracy and assemblies’ overall contiguity. Unfortunately, additional BioNano and Hi-C data without TGS reads are unlikely to help increase the assembly quality because the data are usually ineffective at assisting hybrid assemblers span gaps between existing contigs [14]. To obtain a complete genome, applying LR_Gapcloser, a fast and memory-efficient approach using long reads, would be an excellent choice to close gaps and improve the contiguity of genome assemblies [104].

### Step 10: Genome annotation

Unlike advanced and revolutionized genome sequencing and assembly, getting genome annotation correct remains a challenge. Annotation is the process of identifying and describing regions of biological interest within a genome (both functionally and structurally). While there are various online annotation servers (Table 3), the intended use of the curated data needs to be clearly defined after considering the two options addressed in Step 7 (maximizing in-house workers/collaboration and outsourcing) because the gene-finding problem in eukaryotes is far more difficult than that in prokaryotes such as bacteria. This procedure requires advanced bioinformatics skills, pipelines, and computing resources and consists of three main steps: (1) identifying noncoding regions; (2) identifying coding regions (called gene prediction); and (3) attaching the biological information of these elements.

**Table 3:** Commonly used genome annotation tools and programs.

Name	Official link	Main feature
Online pipeline		
NCBI	https://www.ncbi.nlm.nih.gov/genome/annotation_euk/process/	Eukaryotic genome annotation. An automatic pipeline with flexibility and speed. Good for beginners and easy to use.
	https://www.ncbi.nlm.nih.gov/genome/annotation_prok/standards/	Prokaryotic genome annotation. An automatic pipeline with flexibility and speed. Good for beginners.
Ensembl	http://ensemblgenomes.org/info/data/annotation https://asia.ensembl.org/info/genome/genebuild/assembly.html	Genome annotation. An automatic pipeline for importing external data or using predictive algorithms. Good for beginners and easy to use. Annotation and prediction.
GenSAS	https://www.gensas.org	Integrates with JBrowse and Apollo. An automatic platform and pipeline for genome structural and functional annotation. A user-friendly interactive portal that includes visualization and editing. Good for beginners and easy to use.
GO FEAT	http://computationalbiology.ufpa.br/gofeat/	Genome and transcriptome. A rapid automatic platform for functional annotation and enrichment. A user-friendly portal that can export results in different output formats. Good for beginners and easy to use.
Blast2GO	https://www.blast2go.com	Functional annotation. An automatic platform as a standalone application that has high throughput and is interactive. A user-friendly program with easy start-up and low maintenance. Good for beginners, but the pro version requires a commercial license.
AmiGO	http://amigo.geneontology.org/amigo	GO and GO enrichment analysis. A user-friendly web-based platform. Requires some configuration of public databases with Perl, JavaScript, and Linux for the standalone application. A good web resource for beginners, but local installation requires bioinformatics support.
eggNOG	http://eggnogdb.embl.de/#/app/home	Database of orthologous groups and functional annotation. An automatic platform and pipeline for any genome that scales with speed and flexibility (15 and 2.5 times faster than BLAST and InterProScan, respectively). Requires some configuration of public databases with various computer languages for a standalone application. A good web resource for beginners, but local installation requires bioinformatics support.
KAAS	https://www.genome.jp/tools/kaas/	Ortholog assignment and pathway mapping. An automatic platform but has a limited number of query sequences. A good web resource for beginners, but local installation requires bioinformatics support.
Augustus	http://bioinf.uni-greifswald.de/augustus/	Gene/genome structure and annotation using ab initio and transcript-based prediction. An automatic platform and pipeline for eukaryotic genomes. Requires some configuration of public databases with various computer languages and dependencies for a standalone application. A good web resource for beginners, but local installation requires bioinformatics support.
GAAP	http://GAAP.hallym.ac.kr	A semiautomated genome assembly and annotation pipeline.
Command line interface		
BRAKER	https://github.com/Gaius-Augustus/BRAKER	Gene/genome structure and annotation using a combination of GeneMark-ET, Augustus, and RNA-seq evidence. A fully automated training platform for novel eukaryotic genomes. Requires 2 input files: an RNA-seq alignment file in BAM format and a corresponding genome file in fasta format. Good for intermediate and advanced users due to the requirement of several semi-unsupervised pipelines and dependencies in local installation.
MAKER	https://www.yandell-lab.org/software/maker.html	Gene/genome structure and annotation pipeline. An easy-to-use semiautomatic pipeline for the de novo annotation of newly sequenced genomes for updating existing annotations to reflect new evidence or just to combine annotations, evidence, and quality control statistics for use with other GMOD programs such as G/JBrowse, Chado, and Apollo. Good for intermediate and advanced users due to the requirement of several semi-unsupervised pipelines and dependencies in local installation.
Cufflinks	http://cole-trapnell-lab.github.io/cufflinks/	Transcriptome assembly and differential expression analysis of RNA-seq. A semiautomatic pipeline that includes TopHat (read mapping) and CummeRbund (visualization and exploration). Good for intermediate and advanced users due to the requirement of several pipelines and dependencies in local installation.
StringTie	https://ccb.jhu.edu/software/stringtie/	A fast and highly efficient assembler of RNA-seq alignment that allows users to quantitate full-length transcripts representing multiple splice variants for each gene locus. A semiautomatic pipeline using a BAM alignment input file with RNA-seq read mappings (produced and converted by TopHat, HISAT2, and Samtools). Good for intermediate and advanced users due to the requirement of several pipelines and dependencies in local installation.
GLEAN	https://sourceforge.net/projects/glean-gene/	An unsupervised learning system for gene structure prediction. A semiautomatic pipeline without prior training. Lacks proper documentation and resources to run programs. Might be good for advanced users due to the requirement of several pipelines and dependencies in local installation.
BLAST	https://blast.ncbi.nlm.nih.gov	A specialized algorithm to find regions of local similarity between sequences. A semiautomatic pipeline for understanding biological sequences. A good web resource for beginners, but local installation requires bioinformatics support.
Modeler	https://evidencemodeler.github.io	Software combining ab initio gene predictions and protein/transcript evidence into weighted consensus gene structures. A semiautomatic pipeline with a flexible and intuitive framework for gene structure annotation. Good for intermediate and advanced users due to the requirement of several pipelines and dependencies in local installation.
GSNAP	http://research-pub.gene.com/gmap	A genomic mapping and alignment program for mRNA and ESTs. A semiautomatic pipeline for gene structure annotation. Good for intermediate and advanced users due to the requirement of several pipelines, configurations, and dependencies in local installation.
SNAP	https://github.com/KorfLab/SNAP	Semi-HMM-based nucleic acid parser gene prediction tool. A semiautomatic pipeline for gene structure annotation. Good for intermediate and advanced users due to the requirement of several pipelines, configurations, and dependencies in local installation.
TopHat	https://ccb.jhu.edu/software/tophat/index.shtml	A fast splice junction mapper for RNA-seq. A semiautomatic pipeline that includes Bowtie and HISAT2 (read aligner). Good for intermediate and advanced users due to the requirement of several pipelines and dependencies in local installation.
PASA	https://github.com/PASApipeline/PASApipeline/wiki	Program for assembling spliced alignments for genome annotation and gene structures. A semiautomatic pipeline for gene structure annotation but useful for genome-guided and de novo RNA-seq assemblies to generate a comprehensive transcript database. Good for intermediate and advanced users due to the requirement of several pipelines and dependencies in local installation.
Evigan	http://www.seas.upenn.edu/~strctlrn/evigan/evigan.html	Predicts genes by integrating multiple evidence sources. An automated annotation program that employs a Dynamic Bayesian Network. Model parameters are estimated by the Expectation–Maximization algorithm, thus eliminating the need to curate training data. Good for intermediate users due to the local installation requirement.
Noncoding RNAs		
Ensembl	https://asia.ensembl.org/info/genome/genebuild/ncrna.html	Automatic annotation of noncoding genes but requires registration. A good web resource for beginners.
LncFunTK	http://sunlab.cpy.cuhk.edu.hk/lncfuntk/	Functional annotation of long noncoding RNAs. An easy-to-use automatic pipeline for newly assembled genomes but requires several input files such as expression profiles (GTF format), TF binding profiles (BED format), and miRNA-binding profiles. This is a good web resource for beginners but might be better for intermediate and advanced users due to the requirement of several input files, pipelines, configurations, and dependencies in local installation.
NONCODE	http://www.noncode.org	Database for noncoding RNAs except tRNAs and rRNAs. An automatic pipeline including 6 steps, format normalization (BED or GTF), combination, filtering protein-coding RNA, information retrieval, advanced annotation, and web presentation. This has a good user-friendly web interface for beginners, but it might be better for intermediate and advanced users due to the requirement of several pipelines, configurations, and dependencies in local installation.
deebBase	http://rna.sysu.edu.cn/deepBase/	Small RNAs, lncRNAs, and circular RNAs
lncRNAdb	https://rnacentral.org/expert-database/lncrnadb	A database that provides comprehensive annotations of eukaryotic long noncoding RNAs. An easy-to-use open public resource. An automatic pipeline for single sequences and a semiautomatic pipeline for multiple sequences with bioinformatic scripts. This has a good user-friendly web interface for beginners but it might be better for intermediate and advanced users due to the requirement of several pipelines, configurations, and dependencies in local installation.
Repeat element		
RepeatMasker	http://repeatmasker.org	A program to screen for interspersed repeats and low-complexity DNA sequences. A fast and sensitive semiautomatic pipeline for assembled genomes. Good for intermediate and advanced users due to the requirement of several databases, pipelines, and dependencies in local installation.
RepeatRunner	http://www.yandell-lab.org/software/repeatrunner.html	A CGL-based program that integrates RepeatMasker with blastx to identify repetitive elements. A semiautomatic pipeline for assembled genomes. Good for intermediate and advanced users due to the requirement of several databases, configurations, pipelines, and dependencies in local installation.
RepBase	http://www.girinst.org/repbase/update/index.html	A database of prototypic sequences representing repetitive DNA from different eukaryotic species. A semiautomatic pipeline for genome sequencing projects. This has a good user-friendly web interface for beginners but it might be better for intermediate and advanced users due to the requirement of several pipelines, configurations, and dependencies in local installation.

BAM, binary alignment map; BED, browser extensible data; ESTs, expressed sequence tags; GO, gene ontology; GTF, gene transfer format; HMM, hidden Markov model; RNA-seq, RNA sequencing; TF, transcription factor.

Recent works have described genome annotations well [13,105–109]. However, it is highly recommended that beginners select automatic or semiautomatic annotation methods (including the workflow and guideline in Fig 1) because manual annotation can be very time- and labor-intensive and expensive. Note that while automatic procedures help accelerate the annotation process, they decrease the confidence and reliability of the outcomes because results from different servers and/or databases are often dissimilar [106,110,111]. Furthermore, automatic annotation algorithms, frequently based on orthologs from distantly related model organisms, cannot yet correctly identify all genes within a genome and manual annotation is often necessary to obtain accurate gene models and gene sets [106,110,111]. Thus, a scheme to obtain consensus annotations by integrating different results, a semiautomatic method, is in demand because this could balance automatic and manual approaches, which would increase the reliability of the annotation while accelerating the process [106,110,111]. In general, the identification of noncoding regions includes small and long sequences including repetitive and transposable elements (Fig 1 and Table 3). Despite an explosion of interest in noncoding data and the massive volume of scientific data, selecting the best strategy to annotate and characterize noncoding RNAs is a daunting task because of the strengths and weaknesses of each computational and empirical approach [112]. After screening noncoding regions (e.g., repeat masking and transposable elements), elements of the gene structure (e.g., introns, exons, coding sequences [CDSs], and start and end coordinates) can be predicted for coding regions.

Both ab initio and evidence-based prediction approaches are widely used as each approach has pros and cons. While Augustus and SNAP are the most popular tools for ab initio prediction, they still necessitate the information of the closely related gene and genome model for screening against the newly sequenced genome. By contrast, evidence-based prediction usually uses results obtained by aligning ESTs, protein sequences, and RNA-seq data (results are even better with full-length Iso-Seq data from PacBio or ONT) to a genome assembly as external evidence. Trained gene predictors (training with Augustus and SNAP to obtain more accurate annotation results is highly recommended) can be used in MAKER, BRAKER, and StringTie (Fig 1 and Table 3). When extrinsic evidence from RNA-seq and protein homology information is available, any program/pipeline could be useful for the de novo annotation of novel genomes. In particular, if any RNA-seq data and a genome sequence are available, starting from MAKER and BRAKER over StringTie would be a better choice for a first-time user because MAKER and BRAKER include ab initio prediction (e.g., Augustus training) unlike StringTie (evidence-based prediction only). However, MAKER could be a better choice for updating existing annotations to reflect new evidence. If various gene prediction methods and tools are used to derive the gene structure from a genome, combining these results to obtain the single consensus gene structure via Evidence Modeler, GLEAN, Evigan, or GAAP is essential (Table 3). In particular, BRAKER, StringTie, PASA, and GAAP can update any gene structure annotation by correcting exon boundaries and adding untranslated regions and alternatively spliced models based on assembled transcriptomic data. The evolutionary rapid emergence of new genes (which quickly respond to changing selection pressures) could give rise to orphan genes that might share no sequence homology to genes in closely related genomes [113]. Combining the methods and results (especially MAKER, BRAKER and StringTie) could therefore prove effective in increasing the number and accuracy of annotation predictions assigned to orphan and any other young genes.

Subsequently, functional annotation—the process of attaching biological information to gene or protein sequences—must be performed. This can be carried out through homology search and gene ontology (GO) term mapping. To investigate gene function or predict evolutionary associations, newly assembled sequences should be compared with gene sequences with known functions to find sequences with high homology using BLAST, Cufflinks, TopHat, GSNAP, Blast2GO/OmicsBox (referred to here as Blast2GO), and GAAP (Fig 1 and Table 3). To label more diverse biological information, GO term mapping should be performed, which allows information about gene-related terms and relations between genes to be stored in three categories: biological processes, molecular functions, and cellular components. Mapping is the process of retrieving GO terms associated with hits (mapping sequences) obtained via a previous homology search (mainly BLAST) that are accessible from AmiGO, Blast2GO, GO-FEAT, and eggNOG-Mapper. Starting from Blast2GO would be a practical choice for a complete novice because it has more graphic user interface mode with explanations.

While Fig 1 and Table 3 provide a summary of useful tools with key features, it is highly recommended to be familiar with the regular update of public databases and pipelines. In addition, understanding the performance and capability of various analysis from a detailed comparison and instructions of common features of annotation tools could be a very important factor for a successful genome annotation, structurally [7,111,114–117] and functionally [8,118–123].

### Step 11: Build a searchable and sharable output format

Research papers and data products (researchers are usually required to submit raw sequencing data to appropriate repositories such as Sequence Read Archive [SRA]) are key outcomes of the scientific enterprise, including most successful genome projects. In addition, most genomic projects/data potentially have value beyond their initial purpose but only if shared with the scientific community, including refining assembly and annotation (see Step 12). In recent years, genomic studies have involved complex datasets such that biologists have become “big data practitioners” [124] because of improvements in high-throughput DNA sequencing and cost reductions. As a result, genomic studies have become routine procedures, and there is widespread demand for tools that can assist in the deliberative analytical review of genomic information. What happens to the data after such projects end? In general, data or data management plans have become the central currency of science because open access, open data, and software are critical for advancing science and enabling collaboration across multiple institutions and throughout the world and increasing public awareness [125]. For example, when archiving sequencing data, repositories such as those run by the National Center for Biotechnology Information (NCBI) and European Bioinformatics Institute (EBI) both provide locations for data archiving and encourage a set of practices related to consistent data formatting and the inclusion of appropriate metadata. However, this is a difficult task for an individual research group due to the wide variety of data formats, dataset sizes, data complexity, data use cases, ethical questions, and data collection/storage/sharing practices [124,126–128]. Despite its importance, major barriers remain to sharing data, software, and research products throughout the scientific community because of the difficulties that interdisciplinary and/or translational researchers face when engaging in collaborative research [124,125,127]. To this end, recent works have provided principles that can be applied in genomic data/database projects, including data sharing and archiving via collaborations [124–128].

The following three fundamental questions on this topic should be considered: (1) Do you want to share your data? (2) Do you have enough in-house expertise and infrastructure to maintain and improve the data, including data storage space? (3) Do you want to form internal and external collaborations to increase research productivity? While each research group has different experiences and criteria in collaborations that included data sharing, engaging with multisite collaborations is highly recommended to overcome more pitfalls, including open-ended questions/concerns on genomic data. In addition, sharing open genomic data can easily facilitate reproducibility and repeatability by reusing the same genomic data.

### Step 12: Reach out to the community to refine the assembly and annotation

Dropping whole-genome shotgun sequencing costs and improvements in bioinformatics pipelines and computer capabilities have resulted in the situation where a small lab can undertake genome projects (assembly and annotation), and any organism can become a model species. Ironically, the ease of sequencing and assembly presents another challenge for annotation: contamination of the assembly itself, because errors in assembly can cause errors in the annotation (structural and functional). In addition, it is important to ensure that methods are computationally repeatable and reproducible because there have been numerous reports of instability arising from a mere change of Linux platform, even when using the exact same versions of genomic analysis tools [49]. When including new data, it is also necessary to provide software infrastructure to assist in genomic data updating. Hence, assembled genomes and curated annotations should not and cannot be considered perfect, static, or “final products.” Data must be maintained, refreshed, and updated to ensure their reuse and discovery.

Manual and continuous annotation is critical to achieving reliable gene models and elements; however, this process can be daunting and cost prohibitive for small research communities. While some genome consortia choose to manually review and edit sets via time- and resource-intensive meetings that often require substantial expertise, this still provides opportunities for community building, education, and training. In contrast, for small research groups, it has been proposed that involving undergraduates in community genome annotation consortiums can be mutually beneficial for both education and genomic resources [106]. Alternatively, a collaborative approach using web portals such as Apollo, JBrowse, G-OnRamp (Galaxy-based platform), and ORCAE [129–133] could be sufficiently robust and flexible to enable the members of a group to work simultaneously or at different times to improve the biological accuracy of annotation.

Despite any community-based participatory research approaches taken, the recruitment and coordination of researchers are central to any research project due to the requirement of diverse expertise and collective learning. The ideal way would be to form a national/international collaborative research partnership with diverse organizations [19,134–136]. Alternatively, active promotion via social networks and/or web portal setup could be the most effective way (e.g., Twitter, the Ensemble website, and blogs). Finally, build collective research solidarity by attending conferences would be plausible. There have been previous successful community efforts and involvement in plant (https://nbenth.com/annotator/index, https://solgenomics.net, and https://www.helmholtz-muenchen.de/pgsb) and animal genome projects (http://www.slimsuite.unsw.edu.au/servers/apollo.php, https://bovinegenome.elsiklab.missouri.edu, http://www.gmgi.org/genomics-fish-shellfish, and https://www.sanger.ac.uk/science/data/vertebrate-genomes-sequencing) using the Apollo instance with J Browsers exhibits attractive and effective routes because it is always online, curators can log in whenever they have time, and some minor revisions only require a few seconds (to confirm the gene models). Others require up to 20 minutes to change (UTR boundaries and other structural alterations).

After the initial setup, tasks include maintaining momentum and morale, according to the recommendations described by Pedro and colleagues [137]. Participants bring their own experiences and strengths into this effort. Availability of a training webinar (e.g., https://bit.ly/3gauwn7 and https://bit.ly/36iNQds) would greatly help kick-start the process, alongside a clear set of starting tasks (e.g., a list of genes/families or regions assigned to each curator) and engagement by the community leader. The leader—an enthusiastic champion—can (1) drum up support from their collaborators; (2) fuse community expertise with resources; (3) oversee the project; and (4) act as a liaison between new members wanting to join, the infrastructure provider, and existing annotators. Considering that the collective expertise within a group may be extensive but diverse, it is necessary to standardize the curation for quality control of annotations. To minimize any conflicts that may arise during the annotation process, it is important (1) to have the initial training webinar by laying out clear rules and guidelines; (2) to select a small subset of genes and ask a group of experienced curators to evaluate whether the decisions taken in each case were uniform and sensible; (3) to record webinar training and comments regarding consensus or disagreements for reporting back to the curation team and to edit the tutorial and guidelines; (4) to address this by automated checks and controls (Apollo does not allow this for now or makes it extremely difficult); and (5) to ask multiple reviewers to check each region by reviewing the annotation history in Apollo (labor-intensive method).

Pooling the expertise, resources, and time of active communities could enable a wide range of geographically distance members to participate in a common process, to share and validate the identification of contradictions and the misrepresentation of data on the genomes [137]. After corrections, the datasets (manually verified gene sets) that emerge from these projects can be used to improve the gene sets for closely related genomes and downstream analysis. Dialog and collaboration between community members have an enormous impact. The result of an entire community agreeing on and taking ownership of a single gene set is a major stepping-stone to accelerating the field. Handling the mammoth task of manual gene annotation in the absence of dedicated funding or teams is a great challenge. However, our guidelines could provide a manageable solution for the prospect of this approach becoming commonplace and will continue to engage in community-driven curation efforts.

### Advice for new genomic users to select a basic assembly and annotation pipeline

For a complete novice, our recommendation would be as below (not recommended starting from Illumina only short reads assembly).

1. Pure long-read assembly: PacBio or ONT read sequencing (if combined, PacBio 40X and ONT 25X, or 60X for a single platform) → CANU assembler (alternatively Flye) → BUSCO assessment → Make a decision to add more sequencing data or proceed next step (See Confirm and Refine in Fig 1) → Optional BioNano with RefAligner (still expensive compared to Hi-C data) → Hi-C with 3D-DNA (alternatively HiRise or AllHiC) → Gapclosing with LR_Gapcloser → Arrow with long-read (alternatively Racon) or Pilon polisher with short-read → BUSCO assessment.
2. Hybrid assembly: 10xGC read with Supernova → PacBio or ONT read with CANU (alternatively MaSuRCA) → The rest are same with “Pure-read assembly” from BUSCO assessment to BUSCO assessment.
3. Annotation: NCBI or EBI (a web-based automatic pipeline) → If not, proceed a semiautomatic pipeline starting from structural annotation → RepeatMasker → Ab initio Augustus training with MAKER (alternatively BRAKER) → Evidence-based prediction (RNA-seq) with MAKER (alternatively BRAKER) → Noncoding RNA prediction with NONCODE → Functional annotation with Blast2GO (alternatively AmiGO) → Genome Browser.

## Conclusions

There are no gold standards for genome assembly and annotation. However, the availability of NGS data (particularly TGS data) and their analytical tools has enabled the sequencing of several high-quality genomes of species of importance in aquaculture in recent years. Beginners and small research groups still face challenges, because genome assembly and annotation are usually complex analytical procedures (or pipelines) requiring interdisciplinary collaborations (from biology to computer science) and hefty costs for refining/maintaining the genome. The recommendations addressed here are broad guidelines that could be considered to avoid common pitfalls throughout the whole-genome assembly and annotation process. However, the comprehensive features (e.g., advantages and disadvantages) of each step and/or technology have not been extensively discussed.

Finally, newly emerging technologies and analytical tools could dramatically improve end-to-end genome assemblies and annotations in the future by replacing the years-long efforts of the past with rapid and low-cost solutions. Meanwhile, emphasis should be placed upon the following: First, define the achievable research aim. Second, avoid the trap of trying to secure a perfect/complete genome assembly and annotation, which could lead to a never-ending project. Third, perform assembly and annotation to gain firsthand experience, including in bioinformatics. Fourth, seek internal and external help and advice from experts. Lastly, be open to sharing genomic data to both increase research productivity and promote public awareness.
